# IL-15 Enhances the Persistence and Function of BCMA-Targeting CAR-T Cells Compared to IL-2 or IL-15/IL-7 by Limiting CAR-T Cell Dysfunction and Differentiation

**DOI:** 10.3390/cancers13143534

**Published:** 2021-07-14

**Authors:** Anthony M. Battram, Mireia Bachiller, Victor Lopez, Carlos Fernández de Larrea, Alvaro Urbano-Ispizua, Beatriz Martín-Antonio

**Affiliations:** 1 Department of Hematology, Hospital Clinic, IDIBAPS, 08036 Barcelona, Spain; mbachiller@clinic.cat (M.B.); vlopezd@cnio.es (V.L.); cfernan1@clinic.cat (C.F.d.L.); aurbano@clinic.cat (A.U.-I.); 2 Department of Medicine, University of Barcelona, 08036 Barcelona, Spain; 3 Josep Carreras Leukaemia Research Institute, 08036 Barcelona, Spain; 4 Department of Experimental Hematology, Instituto de Investigación Sanitaria-Fundación Jiménez Díaz, 28040 Madrid, Spain; beatriz.antonio@quironsalud.es

**Keywords:** CAR-T cells, multiple myeloma, IL-2, IL-15, IL-7, BCMA, senescence

## Abstract

**Simple Summary:**

T cells modified with a chimeric antigen receptor (CAR) that targets BCMA, a protein expressed on malignant plasma cells, represent a novel treatment option for multiple myeloma. Despite initially eliminating the disease, the function of BCMA-directed CAR-T cells diminishes within a year of administration, leading to disease relapse. The aim of this research was to alter the cytokines used in the ex vivo expansion of anti-BCMA CAR-T cells, to avoid the development of an unfavorable phenotype that would impair in vivo function. We discovered that CAR-T cells expanded with IL-15 had reduced dysfunction and enhanced persistence compared to those grown with IL-2 or a combination of IL-15 and IL-7, which resulted in longer and improved anti-tumor responses in a mouse model. Therefore, the use of IL-15 alone in place of IL-2 or IL-15/IL-7 should be considered when designing CAR-T cell production protocols, to improve the duration of patient responses.

**Abstract:**

Chimeric antigen receptor (CAR)-T cell immunotherapy has revolutionized the treatment of B-lymphoid malignancies. For multiple myeloma (MM), B-cell maturation antigen (BCMA)-targeted CAR-T cells have achieved outstanding complete response rates, but unfortunately, patients often relapse within a year of receiving the therapy. Increased persistence and reduced dysfunction are crucial features that enhance the durability of CAR-T cell responses. One of the factors that influence CAR-T cell in vivo longevity and loss of function, but which has not yet been extensively studied for BCMA-directed CAR-T cells, are the cytokines used during their production. We here compared the impact of IL-2, IL-15 and a combination of IL-15/IL-7 on the phenotype and function of ARI2h, an academic BCMA-directed CAR-T cell that is currently being administered to MM patients. For this study, flow cytometry, in vitro cytotoxicity assays and analysis of cytokine release were performed. In addition, ARI2h cells expanded with IL-2, IL-15, or IL-15/IL-7 were injected into MM tumor-bearing mice to assess their in vivo efficacy. We demonstrated that each of the cytokine conditions was suitable for the expansion of ARI2h cells, with clear in vitro activity. Strikingly, however, IL-15-produced ARI2h cells had improved in vivo efficacy and persistence. When explored further, it was found that IL-15 drove a less-differentiated ARI2h phenotype, ameliorated parameters related to CAR-T cell dysfunction, and lowered the release of cytokines potentially involved in cytokine release syndrome and MM progression. Moreover, we observed that IL-15 was less potent in inducing T cell senescence and DNA damage accumulation, both of which may contribute to an unfavorable CAR-T cell phenotype. These findings show the superiority of IL-15 to IL-2 and IL-15/IL-7 in the quality of anti-BCMA CAR-T cells, particularly their efficacy and persistence, and as such, could improve the duration of responses if applied to the clinical production of CAR-T cells for patients.

## 1. Introduction

Adoptive immunotherapy using CD19-targeting chimeric antigen receptor (CAR)-modified T cells is now a proven treatment for hematological malignancies such as B-cell acute lymphoblastic leukemia and non-Hodgkin’s lymphoma. For multiple myeloma (MM), BCMA has emerged as the most promising target to which CAR-T cells can be directed [1,2]. Particularly for relapsed/refractory (R/R) MM, which is currently considered incurable [3], novel therapies are urgently required. Various studies have demonstrated impressive response rates for anti-BCMA CAR-T cells (BCMA-CARs) when used to treat R/R MM [4,5,6,7,8], culminating in the recent approval by the FDA of the first BCMA-CAR, idecabtagene vicleucel (ide-cel), for these patients [9].

Despite high rates of initial response following BCMA-CAR treatment, relapses are frequently observed, resulting in average progression-free survival rates of approximately 8–10 months [2,8,10,11]. The most common parameter that has been described to negatively correlate with BCMA-CAR efficacy is a lack of in vivo expansion/persistence [5,6,8,12,13,14]. Several strategies to improve the survival and/or differentiation status, and thus the persistence, of CAR-T cells have been proposed [15]. For BCMA-CARs, some of these approaches for improving the final product have already been studied and implemented, including the use of a 1:1 CD4^+^:CD8^+^ ratio [16], the humanization of the CAR molecule [17], and the inclusion of a phosphoinositide 3-kinase inhibitor in the ex vivo culture [18]. However, the choice of cytokine(s) used to expand BCMA-CARs has not been previously studied in detail.

Currently, high-dose IL-2 is used to expand the BCMA-CAR products that are being investigated in most clinical studies [5,6,8,19]. Although high-dose IL-2 is a strong inducer of T-cell proliferation [20], it additionally promotes terminal differentiation and T-cell exhaustion [21]. IL-15 is an attractive substitute for high-dose IL-2 because they have equal T-cell mitogenic properties [22], but IL-15 does not cause effector T cell exhaustion [21]. Moreover, IL-15 induces the formation of memory cells and enhances the fitness of T cells by delaying their senescence [23,24], which are desirable qualities for CAR-T cells [25]. IL-7 is frequently used in combination with IL-15 because it has been shown to preserve a naïve/stem cell memory phenotype [26]. For anti-CD19 CAR-T cells, replacing IL-2 with IL-15 or IL-15/IL-7 results in improved survival and persistence in pre-clinical models [27,28]. Critical to the observed superior in vivo efficacy was an improved phenotype of the ex vivo expanded CAR-T cells prior to administration in patients, especially an increase in the proportion of memory stem cells and reduced dysfunction [27,28]. Interestingly, Alizadeh et al. showed that anti-CD19 CAR-T cells produced using both IL-15 and IL-7 were inferior to those generated using IL-15 alone [28].

For BCMA-CARs, the optimal cytokine condition for the expansion of CAR-T cell products is currently unknown. Moreover, whether IL-7 is beneficial or not to IL-15-driven CAR-T cell production is an unanswered question. In the present study, we compared how IL-2, IL-15 and a combination of IL-15/IL-7 shaped the phenotype and function of ARI2h cells [17], an in-house BCMA-CAR that is currently undergoing assessment in a clinical trial for R/R MM (NCT04309981). We discovered that ARI2h cells grown in each of the three conditions were comparable in terms of expansion, CAR transduction and in vitro activity, but that IL-15-cultured cells were better than those expanded in IL-15/IL-7 at ameliorating disease progression in a MM xenograft model. Furthermore, we found that IL-15 drove a less-differentiated ARI2h phenotype than when IL-15/IL-7 was used in combination, and additionally, that IL-15-grown ARI2h cells had reduced dysfunction and were more memory stem cell-like than those cultured with IL-2. Finally, we showed that T cell senescence-linked pathways and the release of pathology-associated cytokines are alleviated by IL-15 compared to IL-2.

## 2. Results

### 2.1. IL-15-Grown BCMA-CARs (ARI2h^IL-15^) Express High Surface Levels of ARI2h CAR and Expand Well in Culture

**Figure 1:**
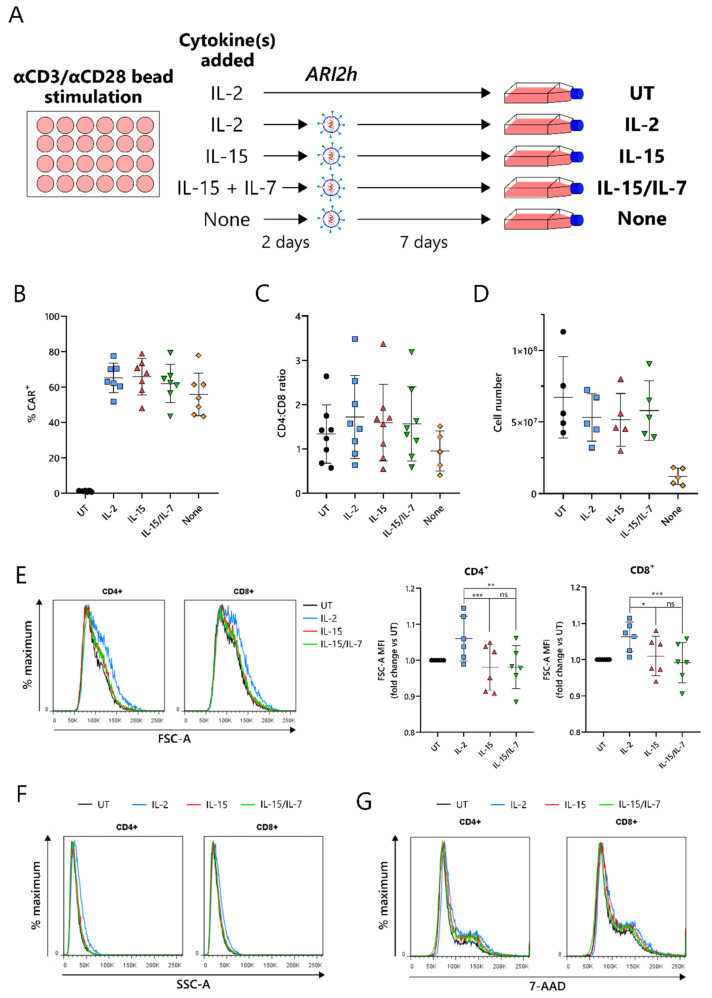
IL-15-cultured ARI2h BCMA-CARs display excellent CAR expression and in vitro expansion. (**A**) Schematic of ARI2h cell culture protocol. (**B**,**C**) Percentage (**B**) and CD4^+^:CD8^+^ ratio (**C**) of CAR^+^ T cells from untransduced (UT) or transduced (ARI2h^IL-2^, “IL-2”; ARI2h^IL-15^, “IL-15”; ARI2h^IL-15/IL-7^, “IL-15/IL-7”, ARI2h^None^, “None”) cultures, analyzed 5–7 days after transduction. (**D**) Cell count of UT and ARI2h cultures, analyzed 7 days after transduction. (**E**) Left—forward scatter (FSC-A) histogram of UT or CAR^+^ CD4^+^ and CD8^+^ day 9 in vitro-cultured T cells, representative of 6 experiments. Right—summary of the FSC-A median fluorescence intensity (MFI) normalized to UT. (**F**) Side scatter (SSC-A) histogram of UT or CAR^+^ CD4^+^ and CD8^+^ day 9 in vitro-cultured T cells, representative of 6 experiments. (**G**) 7-AAD-stained DNA content in UT or CAR^+^ CD4^+^ and CD8^+^ day 9 in vitro-cultured T cells. Images shown are representative histograms (*n* = 4). * *p* < 0.05; ** *p* < 0.01; *** *p* < 0.001; ns, not significant.

Current protocols for the expansion of BCMA-CARs require the use of IL-2 or IL-15/IL-7 to amplify the CAR-T cell number, following an initial CD3-/CD28-mediated T-cell activation. To compare IL-15 expansion with IL-2 and IL-15/IL-7, T cells were cultured with a concentration of IL-2 that was used for preclinical studies of ARI2h, 100 IU/mL [17], and IL-15/IL-7 concentrations that were used in a previous study and that closely match those used in the clinical trial currently ongoing for ARI2h (NCT04309981)—10 ng/mL for both (Figure 1A) [28]. We found that in vitro expansion of ARI2h CAR-T cells with IL-15 (henceforth referred to as ARI2h^IL−15^) did not disrupt expression of the CAR molecule, alter the CD4^+^:CD8^+^ ratio of the CAR^+^ T cells, or impede CAR-T cell expansion, compared to ARI2h CAR-T cells cultured with IL-2 (ARI2h^IL-2^) or IL-15/IL-7 (ARI2h^IL-15/IL-7^) (Figure 1B–D). On the other hand, without adding cytokines to the in vitro culture, ARI2h CAR-T cells (ARI2h^None^) had a lower CD4^+^:CD8^+^ ratio and did not expand to the same extent as ARI2h^IL-15^, ARI2h^IL-2^, or ARI2h^IL-15/IL-7^ (Figure 1C,D).

One of the major differences between IL-2- and IL-15-grown T cells is that IL-2-cultured cells have an increased cell size due to an augmented protein content [22]. In agreement with this, ARI2h^IL-2^ cells were larger and more complex than ARI2h^IL-15^ cells, according to the forward scatter (FSC-A) and side scatter (SSC-A), respectively, of CD4^+^ and CD8^+^ cells, as determined by flow cytometry (Figure 1E,F and Figure S1). A similar DNA content in ARI2h^IL-2^, ARI2h^IL-15^ and ARI2h^IL-15/IL-7^ cells suggested that the difference in cell size was not explained by altered cell cycle progression (Figure 1G). Interestingly, the addition of IL-7 in the expansion of ARI2h^IL-15/IL-7^ made no difference to the cell size or cell cycle progression when compared to ARI2h^IL-15^ cells.

### 2.2. ARI2h^IL-15^ Functional Responses Are Indistinguishable from Those Exhibited by ARI2h^IL-2^ or ARI2h^IL-15/IL-7^

**Figure 2:**
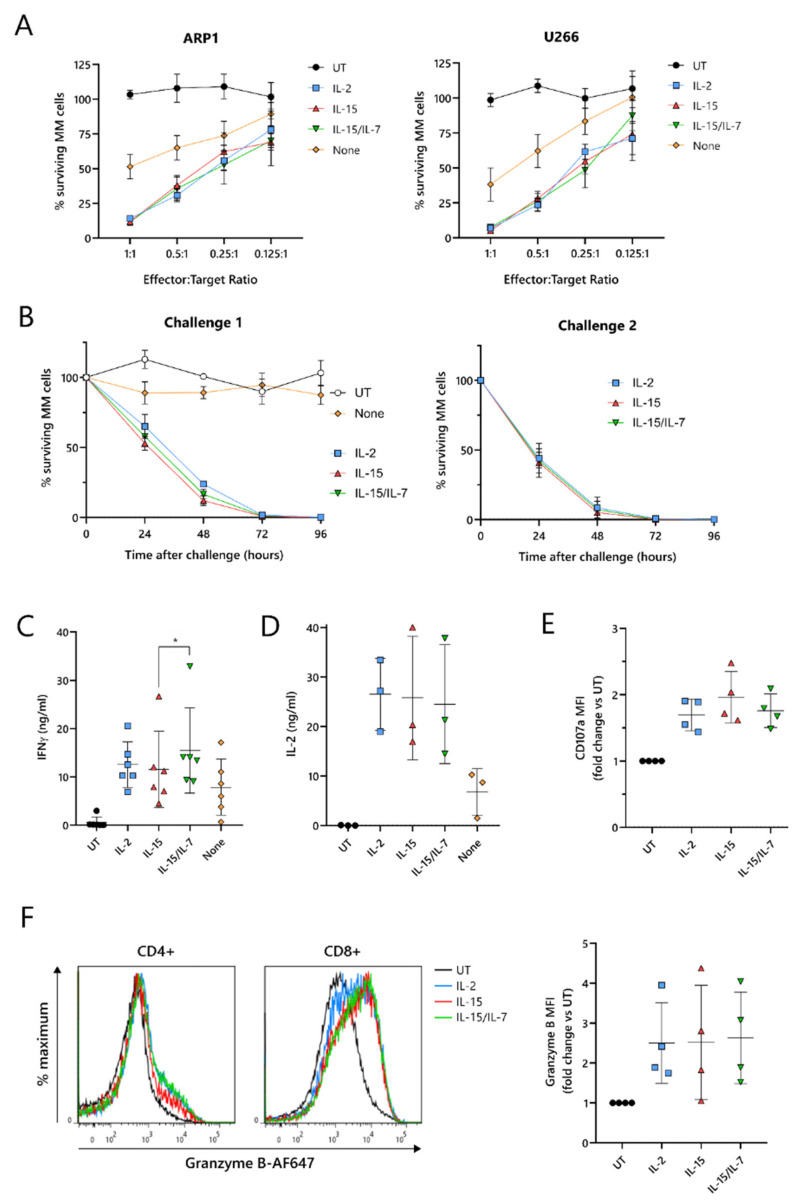
IL-15-grown ARI2h BCMA-CARs are highly functional. UT and ARI2h-transduced (ARI2h^IL-2^, ARI2h^IL-15^, ARI2h^IL-15/IL-7^, ARI2h^None^) T cells were co-cultured with ARP-1 cells for 24 h (**A**), 96 h (**B**) or 6 h (**C**–**F**) or U266 cells for 24 h (**A**). (**A**) Survival of GFP-ffLuc-expressing ARP-1 (left) or U266 (right) multiple myeloma (MM) cell lines, following a 24-hour co-culture with UT or ARI2h cells at the indicated T cell:tumor cell line (effector:target) ratios (*n* = 5). (**B**) Survival of GFP-ffLuc-expressing ARP-1 cells following a 96-hour co-culture with UT or ARI2h cells, measured every 24 h. Graphs show the results of a first co-culture (left) and a second challenge (right) of ARI2h^IL-2^, ARI2h^IL-15^ and ARI2h^IL-15/IL-7^ cells with fresh ARP-1 cells. All challenges were performed at a 0.125:1 effector:target ratio (*n* = 3). (**C**,**D**) Levels of released IFNγ (**C**) and IL-2 (**D**), as measured by ELISA. (**E**) Summary of the surface expression of CD107a on CD8^+^ (UT) or CD8^+^ CAR^+^ T cells, based on the MFI and normalized to UT. (**F**) Left—representative histograms showing granzyme B staining in UT or CAR^+^ CD4^+^ and CD8^+^ T cells. Right—summary of the granzyme B expression in CD8^+^ (UT) or CD8^+^ CAR^+^ T cells, based on the MFI and normalized to UT. * *p* < 0.05.

To test their cytotoxic function, ARI2h CAR-T cells expanded with IL-2, IL-15, IL-15/IL-7, or no cytokine were co-cultured with two luciferase-expressing MM cell lines, ARP-1-GFP and U266-GFP. The cytotoxicity exhibited by ARI2h^IL−15^ against these tumor cells was similar to ARI2h^IL-2^ and ARI2h^IL-15/IL-7^ at effector:target (E:T) ratios ranging from 1:1 to 0.125:1 (Figure 2A). Furthermore, ARI2h^IL-15^, ARI2h^IL-2^ and ARI2h^IL-15/IL-7^ fully eliminated ARP-1-GFP cells within 96 h, even at a low E:T ratio, but, in contrast, ARI2h^None^ cells exhibited little to no function (Figure 2B—left). When ARI2h^IL-15^, ARI2h^IL-2^ and ARI2h^IL-15/IL-7^ were rechallenged with tumor cells (Figure S2A), they exhibited a more efficient cytotoxic functionality than after the first challenge (Figure 2B) but expanded slightly less (Figure S2B). For ARI2h^IL-15^, ARI2h^IL-2^ and ARI2h^IL-15/IL-7^, the proportion of CAR^+^ cells increased after each challenge (Figure S2C), as previously reported for ARI2h^IL-2^ [17], and the CD4^+^:CD8^+^ ratio of CAR^+^ T cells decreased (Figure S2D).

Critical to the function of CAR-T cells is their ability to produce cytokines and effector molecules in response to target tumor cells. When exposed to MM cells for 6 h, ARI2h^IL−15^ cells produced a high quantity of IFNγ, similar to that made by ARI2h^IL−2^ cells, but less than that released by ARI2h^IL-15/IL-7^ cells (Figure 2C and Figure S2E). IL-2, which is critical for effector T cell proliferation and function [29], was also produced at high concentrations by ARI2h cells expanded in each of the cytokine conditions (Figure 2D and Figure S2F). The cytokine(s) used to expand the ARI2h cells also had little effect on the ARP-1-mediated degranulation, as measured by surface levels of CD107a (Figure 2E), or the production of cytotoxic molecule granzyme B (Figure 2F). Basal expression of granzyme B was similar across all ARI2h CARs, suggesting a similar ability to make granzyme B in response to cytokine signaling (Figure S2G).

Put together, these results show that, in vitro, ARI2h^IL-15^ cells are not inferior to ARI2h^IL-2^ or ARI2h^IL-15/IL-7^ in terms of the production of functional CAR-T cells. These findings also confirm that the presence of a γ-chain cytokine in the expansion of ARI2h is critical.

### 2.3. ARI2h^IL-15^ Has a Superior In Vivo Function Than ARI2h^IL-15/IL-7^

**Figure 3:**
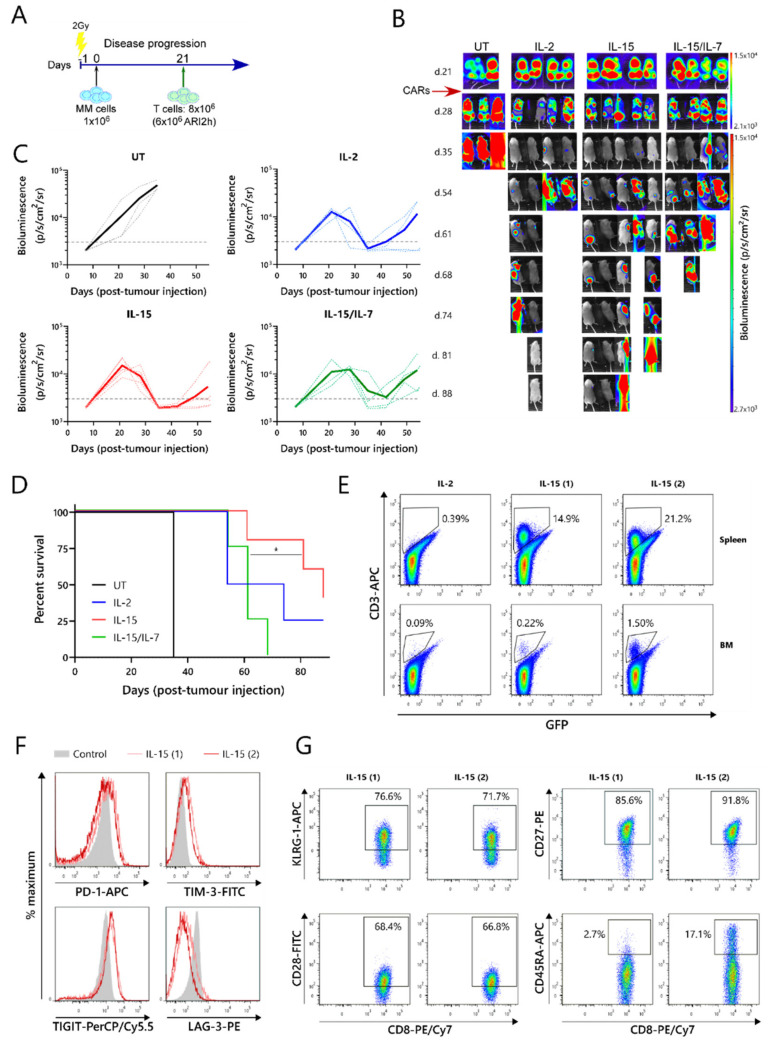
ARI2h BCMA-CARs expanded in IL-15 have superior in vivo function. (**A**) Schematic of in vivo experimental design—mice were injected with 1 × 10^6^ GFP-ffLuc-expressing ARP-1 cells and, three weeks later, were transfused with 8 × 10^6^ T cells from UT or ARI2h-transduced (ARI2h^IL-2^, ARI2h^IL-15^ and ARI2h^IL-15/IL-7^) cultures. (**B**) Tumor progression was measured by taking weekly bioluminescence images. Figures on the left of the image show the number of days following tumor cell infusion. (**C**) Quantification of bioluminescence. Dotted lines show data from individual mice and solid lines show the average from all mice in that group. The dashed line at 3000 p/s/cm^2^/sr is included for comparison purposes. (**D**) Overall survival of mice from each group. (**E**) Frequency of anti-human CD3^+^ ARI2h cells in the spleen and bone marrow (BM) of all surviving mice (IL-15 (1) and IL-15 (2) refer to the first and second mice from the IL-15 group, respectively). Numbers indicate the percentage of cells found within the ARI2h gate. (**F**,**G**) Flow cytometry analysis of exhaustion (**F**) and senescence (**G**) markers on CD8^+^ ARI2h cells found in the spleen of the surviving mice from the IL-15 group. “Control” indicates unstimulated human T cells (**F**). Numbers indicate the percentage of cells found within the gate shown (**G**). * *p* < 0.05.

The relative efficacies of ARI2h^IL−15^, ARI2h^IL−2^ and ARI2h^IL-15/IL-7^ were further examined in an in vivo murine model of MM. For this experiment, NOD-SCIDIL2gc^−^/^−^ mice that were previously engrafted with ARP-1-GFP cells until developing a high tumor burden were injected with untransduced (UT) T cells, ARI2h^IL-15^, ARI2h^IL-2^, or ARI2h^IL-15/IL-7^ (Figure 3A,B). All of the mice that received ARI2h exhibited an initial decline in their tumor levels, whereas the disease continued to progress in animals that received UT T cells (Figure 3B,C). However, many of the mice treated with ARI2h eventually experienced tumor regrowth, with those treated with ARI2h^IL-15/IL-7^ relapsing more quickly than those injected with ARI2h^IL-15^ or ARI2h^IL-2^ (Figure 3B,C). Correspondingly, the overall survival time of mice from the ARI2h^IL-15^ group was significantly longer than that of mice from the ARI2h^IL-15/IL-7^ set (Figure 3D). Interestingly, the two surviving mice from the ARI2h^IL-15^ group did begin to develop new tumors four weeks after the CAR-T cell infusion, but these were cleared without any reinfusion of CAR-T cells (Figure 3B—IL-15 mice 1 and 2 from day 54 onward). Strikingly, these surviving ARI2h^IL-15^-treated animals were the only mice in which CAR-T cells were found in substantial numbers in the spleen and bone marrow following sacrifice (Figure 3E and Figure S3A).

Further analysis of the ARI2h^IL-15^ cells from these mice showed that the CD8^+^ cells expressed intermediate levels of the exhaustion markers PD-1, TIM-3 and TIGIT, but low levels of LAG-3 (Figure 3F). The CD4^+^ ARI2h^IL-15^ cells displayed a similar exhaustion phenotype (Figure S3B). To characterize the dysfunction of the ARI2h^IL-15^ cells in more detail, the expression of cell surface markers of T cell senescence, namely, CD28, CD27, KLRG-1 and CD45RA [30], were analyzed. Although over 70% of the CD8^+^ cells expressed KLRG-1, they were predominantly CD28^+^CD27^+^CD45RA^-^, suggesting that they had a central memory phenotype and were not late-differentiated senescent cells (Figure 3G). Similarly, the CD4^+^ ARI2h^IL-15^ cells were mostly KLRG-1^+^ and CD28^+^CD45RA^-^, but they did not express CD27 (Figure S3C). Further analysis of the memory phenotype revealed that the majority of ARI2h^IL-15^ cells, especially CD8^+^, were CCR7^-^CD45RA^-^ effector memory cells (Figure S3D).

Overall, these data show that ARI2h^IL-15^ CAR-T cells have enhanced persistence and are superior to ARI2h^IL-15/IL-7^ CAR-T cells at preventing or reducing tumor growth in a MM murine model. In addition, ARI2h^IL-15^ cells did not express LAG-3, an important marker of exhaustion, and did not exhibit a senescent phenotype.

### 2.4. IL-15-Cultured CAR-T Cells Have a More Differentiated Phenotype When Additionally Expanded with IL-7

**Figure 4:**
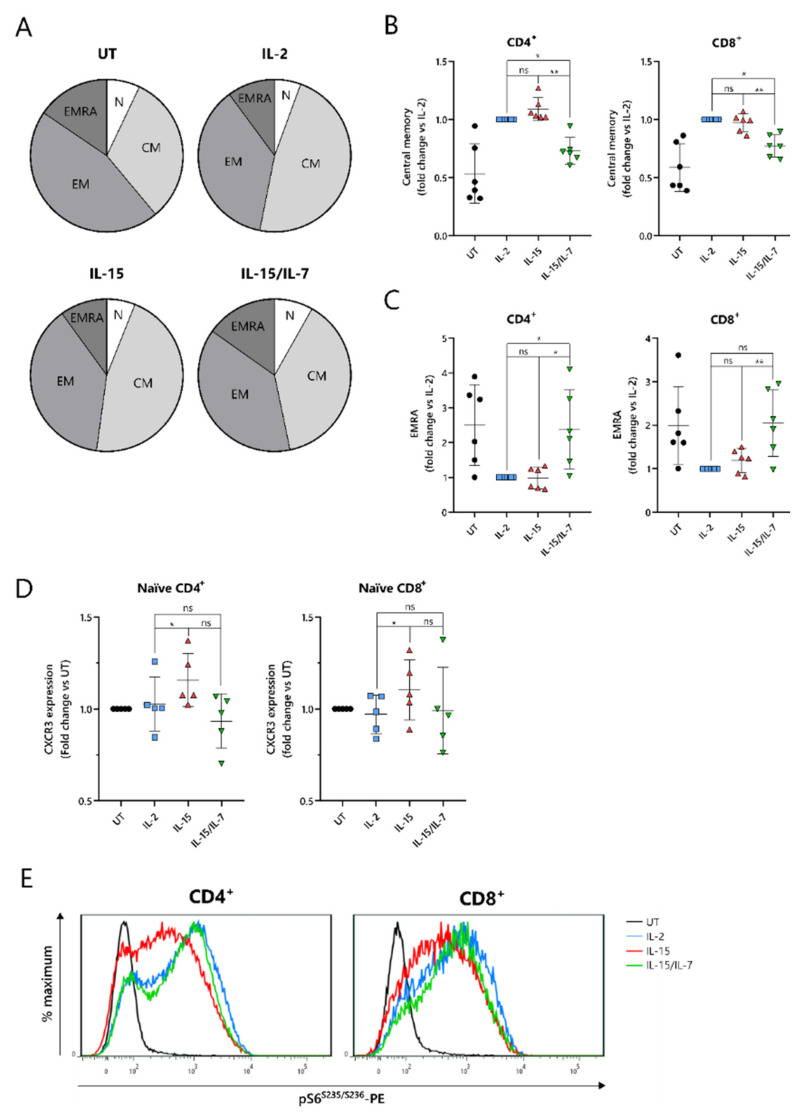
IL-7 drives a more differentiated memory phenotype in ARI2h BCMA-CARs cultured with IL-15. (**A**) Pie charts showing a summary of phenotypes of in vitro-expanded CD8^+^ (UT) and CD8^+^CAR^+^ (ARI2h^IL-2^, ARI2h^IL-15^, ARI2h^IL-15/IL7^) T cells. N—naïve (CCR7^+^CD45RA^+^), CM—central memory (CCR7^+^CD45RA^−^), EM—effector memory (CCR7^-^CD45RA^−^), EMRA—effector memory CD45RA^+^ (CCR7^-^CD45RA^+^). *n* = 6. (**B**,**C**) Relative frequency of T cells with a central memory (**B**) or EMRA (**C**) phenotype within the CD4^+^ and CD8^+^ (UT) or CAR^+^ CD4^+^ and CD8^+^ (ARI2h^IL-2^, ARI2h^IL-15^ and ARI2h^IL-15/IL-7^) cultures, normalized to ARI2h^IL-2^. (**D**) CXCR3 expression on naïve-phenotype CD4^+^ and CD8^+^ (UT) or CAR^+^ CD4^+^ and CD8^+^ (ARI2h^IL-2^, ARI2h^IL-15^ and ARI2h^IL-15/IL-7^) T cells, based on the MFI of the staining and normalized to UT T cells. E) Phosphorylation of the ribosomal S6 protein on serine 235/serine 236 in UT or CAR^+^ CD4^+^ and CD8^+^ T cells after a 6-hour challenge with ARP-1 cells. Images shown are representative histograms (*n* = 4). The statistics for panels B–C were performed on raw data. * *p* < 0.05; ** *p* < 0.01; ns, not significant.

To understand the improved in vivo efficacy of ARI2h^IL-15^ compared to ARI2h^IL-15/IL-7^, we explored the phenotype of the cells following nine days of in vitro culture. The cytokine used for the in vitro proliferation of T cells influences their memory phenotype, which in turn, alters their longevity and functional responses. Indeed, for CD19-directed CAR-T cells, an increase in memory-like cells has been linked with improved anti-tumor function and the ability to maintain proliferation in a host [31]. Interestingly, CD8^+^ ARI2h CAR-T cells were less differentiated than UT CD8^+^ T cells, regardless of the cytokine that was added to the culture, displaying a lower percentage of cells with an effector memory (CD45RA^-^CCR7^-^) or effector (CD45RA^+^CCR7^−^) phenotype (Figure 4A and Figure S4A). CD8^+^ ARI2h^IL-15^ and ARI2h^IL-2^ T cells had a remarkably similar overall percentage of memory/effector phenotype cells, but the makeup of CD8^+^ ARI2h^IL-15/IL-7^ cells was different (Figure 4A). When explored in more detail, both CD4^+^ and CD8^+^ ARI2h^IL-15/IL-7^ cells had a lower proportion of central memory (CD45RA^-^CCR7^+^) phenotype cells than ARI2h^IL-15^ (Figure 4B), while exhibiting a higher fraction of effector phenotype cells (Figure 4C). CD4^+^ ARI2h^IL-15/IL-7^ cultures had more cells with a naïve (CD45RA^+^CCR7^+^) phenotype, but within the CD8^+^ population, ARI2h^IL-2^, ARI2h^IL-15^ and ARI2h^IL-15/IL-7^ naïve phenotype cells were found at similar percentages (Figure S4B).

Another indicator of low differentiation and high self-renewal capacity within a T cell population is the presence of memory stem cells, which can be identified as a subset of the CD45RA^+^CCR7^+^ population that additionally express the chemokine receptor CXCR3 [32]. The expression of CXCR3 was higher within CD45RA^+^CCR7^+^ ARI2h^IL-15^ cells than ARI2h^IL-2^ cells (Figure 4D), both for CD4^+^ and CD8^+^ T cells, suggesting an increased proportion of stem-like cells.

When CAR-T cells are administered to cancer patients and they encounter tumor cells, they differentiate further along either an effector or memory pathway. mTORC1 activity is critical to promoting the effector differentiation lineage of T cells, and as such, reduced mTORC1 activation is thought to be favorable for durable responses. We found that MM cell-induced mTORC1 activity, as measured by phosphorylation of the downstream ribosomal S6 protein, was lower in ARI2h^IL-15^ cells than in either ARI2h^IL-2^ or ARI2h^IL-15/IL-7^ cells, which exhibited similar levels of S6 protein phosphorylation (Figure 4E). This difference was dependent on mTORC1 activity as it was blocked by the mTORC1-specific inhibitor rapamycin (Figure S4C), and moreover, it was not caused by an overall decrease in cell activation, as the induction of CD69 expression was the same in ARI2h CAR-T cells from all groups (Figure S4D). Interestingly, basal mTORC1 activity, in the absence of MM cells, was higher in ARI2h^IL-2^ cells compared to ARI2h^IL-15^ or ARI2h^IL-15/IL-7^, although many cells demonstrated low mTORC1 signaling (Figure S4E).

Thus, these data provide evidence that the addition of IL-15 alone is the best in vitro condition in which to produce ARI2h cells that are less differentiated and have more memory stem-like cells.

### 2.5. ARI2h^IL-15^ Cells Have Reduced Expression of LAG-3 and Augmented Levels of CD27 Compared to ARI2h^IL-2^

**Figure 5:**
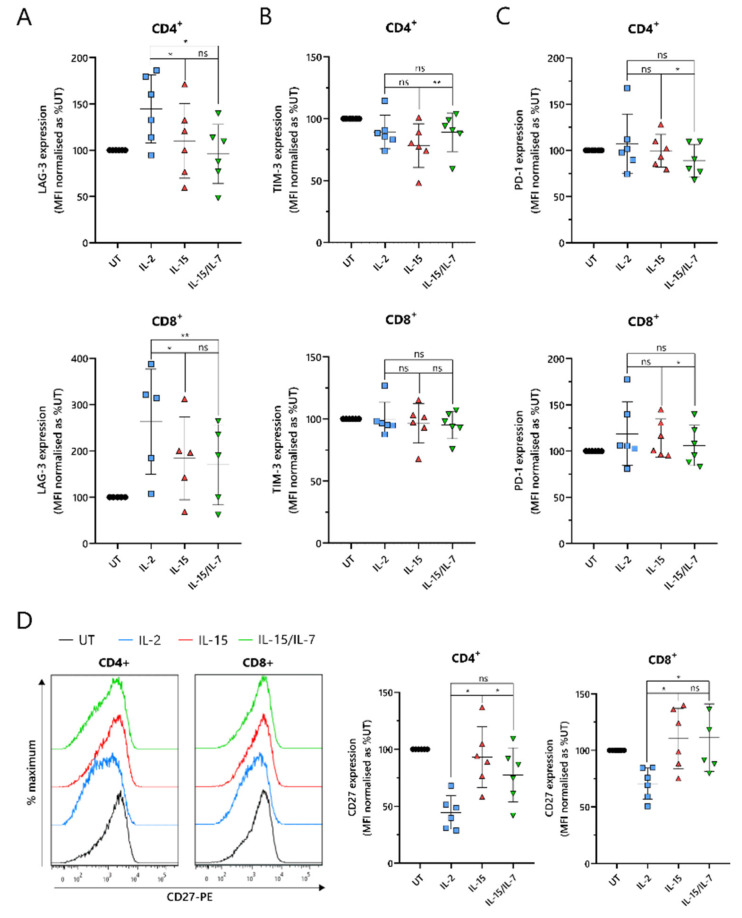
Expression of T-cell dysfunction markers on ARI2h BCMA-CARs. Summary of the surface expression of LAG-3 (**A**), TIM-3 (**B**), PD-1 (**C**) and CD27 (**D**—right) on day 9 in vitro-cultured CD4^+^ and CD8^+^ (UT) or CAR^+^ CD4^+^ and CD8^+^ (ARI2h^IL-2^, ARI2h^IL-15^ and ARI2h^IL-15/IL-7^) T cells, based on the MFI of the staining and normalized to UT. Representative histograms of CD27 staining are also shown (**D**—left) (*n* = 6). * *p* < 0.05; ** *p* < 0.01; ns, not significant.

An important indicator of CAR-T cell efficacy is the expression of exhaustion markers that contribute to T cell dysfunction. In particular, PD-1, LAG-3 and TIM-3 have been proposed as the most significant markers. ARI2h^IL-15^ and ARI2h^IL-15/IL-7^ expressed reduced levels of LAG-3 compared to ARI2h^IL-2^ in both CD4^+^ and CD8^+^ cells (Figure 5A). Although LAG-3 expression was similar on ARI2h^IL-15^ and ARI2h^IL-15/IL-7^ cells, levels of TIM-3 were lower on ARI2h^IL-15^ CD4^+^ cells (Figure 5B). However, PD-1 expression was marginally but significantly lower on ARI2h^IL-15/IL-7^ cells (Figure 5C), suggesting an overall similar exhaustion phenotype between ARI2h cells of the IL-15 and IL-15/IL-7 groups. Another immune checkpoint, TIGIT, inhibits endogenous T cell anti-MM activity, due to the high levels of TIGIT ligands expressed on malignant plasma cells in the BM [33], but expression of this receptor was not different on ARI2h^IL-15^, ARI2h^IL-2^ or ARI2h^IL-15/IL-7^ cells (Figure S5A).

A further source of T cell dysfunction is the loss of expression of costimulatory molecules, particularly CD27 and CD28. CD27 expression was elevated on ARI2h^IL-15^ cells compared to ARI2h^IL-2^ cells, particularly in CD4^+^ cells, where ARI2h^IL-15^ cell CD27 levels were also significantly greater than those on ARI2h^IL-15/IL-7^ cells (Figure 5D). However, on CD8^+^ cells, CD27 expression was similar on ARI2h^IL-15^ and ARI2h^IL-15/IL-7^ cells. Interestingly, CD4^+^CAR^+^ ARI2h^IL-2^ CD27 levels were less than half that seen on IL-2-cultured UT CD4^+^ T cells (Figure 5D). Likewise, CD28 expression was lower on ARI2h^IL-2^ cells compared to UT T cells, but there was no significant difference in CD28 levels between ARI2h^IL−2^ and ARI2h^IL-15^ or ARI2h^IL-15/IL-7^ (Figure S5B).

Altogether, ARI2h^IL-15^ and ARI2h^IL-15/IL-7^ cells exhibited a less dysfunctional phenotype than ARI2h^IL-2^ cells.

### 2.6. ARI2h^IL-15^ Cells Have Reduced DNA Damage Compared to ARI2h^IL-2^

**Figure 6:**
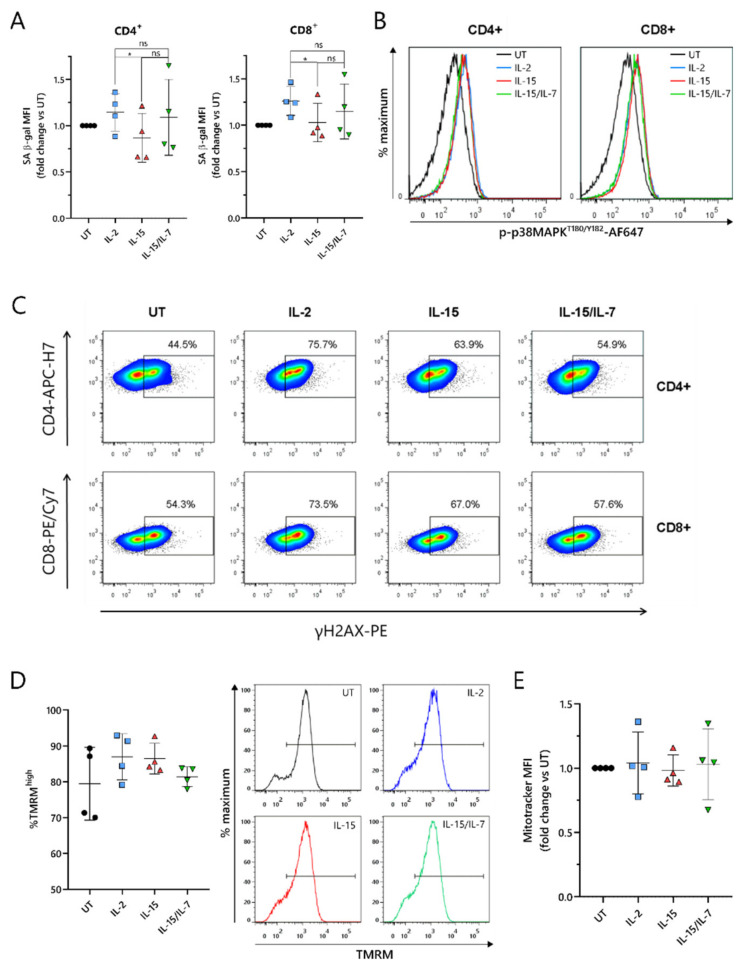
IL-15 induces lower levels of DNA damage in ARI2h BCMA-CARs compared to IL-2 while maintaining high mitochondrial function. (**A**) Summary of the senescence-associated β-galactosidase (SA-β-gal) activity of day 9 in vitro-cultured UT or CAR^+^ CD4^+^ and CD8^+^ T cells, based on the MFI of the signal and normalized to UT. (**B**,**C**) Phosphorylation of p38 MAPK on threonine 180/tyrosine 182 (**B**) or γH2AX expression (**C**) in UT or CAR^+^ CD4^+^ and CD8^+^ day 9 in vitro-cultured T cells. Shown are representative histograms (**B**) (*n* = 4) or representative FACS plots in which the numbers/gates show the percentage of γH2AX^+^ cells based on an FMO control (**C**) (*n* = 4). (**D**) Left—Relative frequency of hyperpolarized (TMRM^high^) cells within the populations of CD8^+^ (UT) or CAR^+^ CD8^+^ (ARI2h^IL-2^, ARI2h^IL-15^ and ARI2h^IL-15/IL-7^) day 9 in vitro-cultured T cells. Right—Representative histograms (*n* = 4). Gates show hyperpolarized (TMRM^high^) cells. (**E**) Summary of the MitoTracker Deep Red FM staining in UT CD8^+^ (UT) or CAR ^+^ CD8^+^ day 9 in vitro-cultured T cells, based on MFI and normalized to UT. * *p* < 0.05; ns, not significant.

As the loss of CD27 expression is a marker of senescence in T cells [34], this was explored further. T cell senescence is a phenomenon that is associated with aging, and naturally occurs due to multiple rounds of replication and telomere loss, but it can also be induced or accelerated by external factors such as nutrient deprivation or the presence of certain metabolites [30]. To investigate whether ARI2h CAR-T cells expanded in IL-2, IL-15 or IL-15/IL-7 exhibited signs of altered senescence, the widely used senescence biomarker, senescence-associated β-galactosidase (SA-β-gal) activity, was analyzed. ARI2h^IL-15^ cells showed reduced SA-β-gal activity compared to ARI2h^IL-2^ cells (Figure 6A), suggesting that ARI2h^IL-2^ cells may be more senescent. Interestingly, this difference was not due to altered p38 MAPK signaling (Figure 6B and Figure S6A), a pathway that is often perturbed in senescent T cells.

We hypothesized that the observed divergences in T cell dysfunctional phenotype between ARI2h^IL-15^ and ARI2h^IL-2^ could be derived from a difference in DNA damage accumulation. Indeed, we found that the proportion of ARI2h^IL-15^ cells that were positive for H2AX phosphorylation (γH2AX) was lower than for ARI2h^IL-2^ cells (Figure 6C and Figure S6B), thus showing that IL-15 causes fewer CAR-T cells to engage in a DNA damage response (DDR). Interestingly, there was a large difference between the proportion of γH2AX^+^ cells in the ARI2h^IL-2^ group compared to UT T cells, indicating that the presence of the CAR induces a strong DDR.

Another hallmark of senescence in T cells is mitochondrial dysfunction and reduced mitochondrial content. Based on the incorporation of tetramethyl rhodamine methyl ester (TMRM), which demonstrates mitochondrial transmembrane potential, ARI2h^IL-2^, ARI2h^IL-15^ and ARI2h^IL-15/IL-7^ all had a high proportion of hyperpolarized cells, suggesting a similar mitochondrial fitness (Figure 6D and Figure S6C). In addition, the uptake of MitoTracker Far Red dye was similar for ARI2h CAR-T cells grown in all three cytokine conditions, suggesting that there was no difference in mitochondrial mass (Figure 6E and Figure S6D,E).

Taken together, these data confirm that ARI2h^IL-2^ exhibits a more senescent phenotype compared to ARI2h^IL-15^, which is associated with increased DNA damage, but not changes in p38 MAPK signaling or mitochondrial function.

### 2.7. ARI2h^IL-15^ Cells Secrete Lower Levels of Cytokines That Are Related to CAR-T Cell Toxicities or MM Progression

A major unresolved issue with CAR-T cell therapy is the triggering of cytokine-release syndrome (CRS), which is the most frequent toxicity associated with this treatment. CRS is caused by cytokines and chemokines that are secreted by activated CAR-T cells, as well as by pro-inflammatory molecules produced by other immune cells, such as macrophages, that are inadvertently stimulated by the CAR-T-cell-derived cytokines/chemokines. To understand whether ARI2h^IL-15^ produced reduced levels of CRS-related molecules, the supernatant of CAR-T cell/ARP-1 cell co-cultures was analyzed for the presence of 34 different cytokines and chemokines. As a control, IFNγ and IL-2 were included in this panel, as they had previously been analyzed by ELISA (Figure S7A,B and Figure 2C,D).

**Figure 7:**
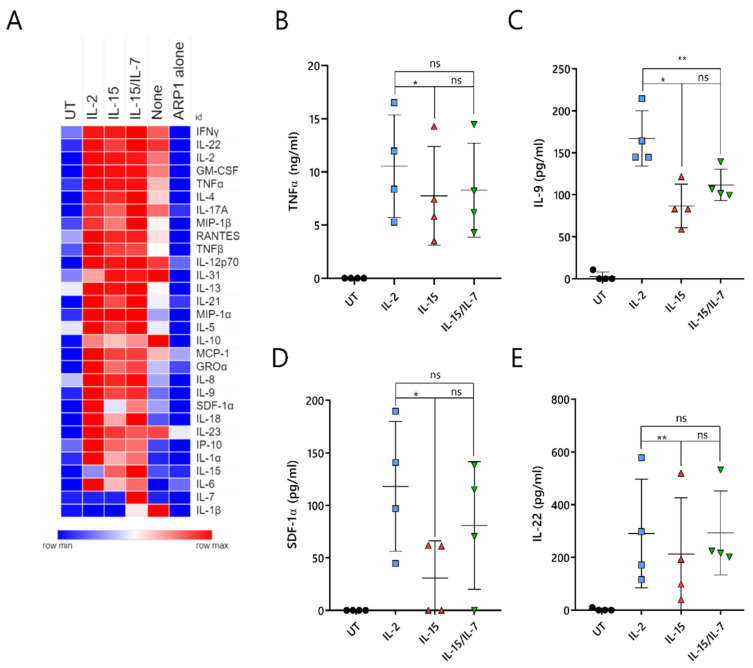
IL-15-grown ARI2h BCMA-CARs produce lower amounts of pathology-related cytokines. UT or ARI2h-transduced (ARI2h^IL-2^, ARI2h^IL-15^, ARI2h^IL-15/IL-7^, ARI2h^None^) T cells, or media (“ARP1 alone”), were co-cultured with ARP-1 cells for 6 h. (**A**) Heat map showing the relative secretion of a panel of cytokines and chemokines (shown on the right-hand side) as analyzed using a ProcartaPlex Multiplex Immunoassay kit (*n* = 4, UT, IL-2, IL-15 and IL-15/IL-7 groups; *n* = 2, “None” group). The relative abundance of each molecule in the analyzed supernatant is shown on a scale of blue (low) to red (high). (**B**–**E**) Individual quantification of TNFα (**B**), IL-9 (**C**), SDF-1α (**D**) and IL-22 (**E**) levels from panel A. * *p* < 0.05; ** *p* < 0.01; ns, not significant.

Overall, ARI2h^None^ cells produced very low levels of almost all analyzed molecules, further highlighting the fact that the addition of IL-2 or IL-15 is critical to the generation of functional ARI2h cells (Figure 7A). Cytokine/chemokine production was similar for ARI2h^IL-2^, ARI2h^IL-15^ and ARI2h^IL-15/IL-7^, although some differences were observed. The release of TNFα, an important macrophage-activating molecule that is associated with CRS severity in MM patients treated with anti-BCMA CAR-T cells [5,13], was reduced in ARI2h^IL-15^ compared to ARI2h^IL-2^ (Figure 7B). In addition, ARI2h^IL-15^ secreted reduced levels of IL-9, SDF-1α and IL-22 compared to ARI2h^IL-2^ cells (Figure 7C–E). All of these are associated with MM progression; high IL-9 levels are related to resistance to proteasome inhibitor therapy, SDF-1α activates MM cells, and an increase in IL-22-producing T_H_22 cells is associated with a poor prognosis for MM patients [35,36,37].

In summary, ARI2h^IL-15^ cells produce high levels of effector cytokines/chemokines when activated by tumor cells, but they release lower levels of secreted molecules that are associated with deleterious effects, such as CRS.

## 3. Discussion

BCMA-directed CAR-T cells are emerging as a promising therapy for incurable R/R MM. Although anti-BCMA CAR-T cells have demonstrated excellent anti-cancer efficacy, a lack of CAR-T cell persistence, resulting in further relapse, is a major shortcoming [8]. The development of a suboptimal phenotype following in vitro CAR-T cell culture is impacted by the cytokine used for their expansion [27,28]. In this report, we show that IL-15 did not adversely affect cell expansion, CAR transduction, or in vitro anti-MM cell activity, compared to IL-2 or a combination of IL-15 and IL-7. Indeed, we conclude that IL-15 alone is the optimal cytokine condition in which to expand ARI2h cells. IL-15 demonstrated superior in vivo function and generated less-differentiated CAR-T cells than IL-15/IL-7 combined, and moreover, IL-15 promoted a more stem cell-like phenotype, reduced dysfunction and caused lower production of cytokines associated with CRS or MM progression compared to IL-2.

It is well established that less-differentiated cells with a more stem cell-like phenotype make for better CAR-T cell products. Many studies have shown that IL-2 expansion induces fewer cells with a naïve or stem cell memory-like phenotype than IL-15 or IL-15/IL-7 [27,28,38]. Our data confirm that IL-2-grown CARs had fewer naïve CD4^+^ cells than IL-15/IL-7-grown CARs, but in contrast, no difference was observed with CD8^+^ cells. This could be explained by the difference in length of ex vivo culture between our study and others. We cultured the CAR-T cells for 9 days to correspond with the protocol used for the clinical-grade production of ARI2h cells [17], whereas in many other studies, CAR-T cells are expanded for two weeks or more. In terms of the stem cell memory phenotype, ARI2h^IL-15^ naïve cells presented the highest expression of the chemokine receptor CXCR3. Furthermore, the addition of IL-7 to IL-15 cultures reduced the proportion of ARI2h cells with a central memory phenotype and increased that of effector cells. Put together with the in vivo results, which showed that ARI2h^IL-15/IL-7^ was an inferior murine therapeutic compared to ARI2h^IL-15^, our data suggest that IL-7 is detrimental to the production of ARI2h cells. Correspondingly, a recent publication found that IL-15-expanded CD19-CAR-T cells demonstrated enhanced anti-tumor efficacy compared to those cultured with IL-15/IL-7 [28]. We believe that the adverse effects caused by IL-7 on CAR-T cell activity is an interesting observation that warrants further investigation.

Another cell surface protein that is a marker of memory cells and self-renewal is CD27. We observed that CD27 was dramatically higher on the surface of ARI2h^IL-15^ cells compared to ARI2h^IL-2^. This is of particular interest because CD27 expression, in the form of CD27^+^CD45RO^-^ cells, was one of the few parameters associated with CART-BCMA efficacy in an R/R MM clinical study [6]. CD27 expression is also associated with T cell dysfunction—lack of CD27 and CD28 expression on T cells is a sign of senescence [30]. We found that, despite CD28 levels being similar, SA β-gal activity and γH2AX levels were lower in ARI2h^IL-15^ cells compared to ARI2h^IL-2^, suggesting reduced senescence and DDR engagement. Based on these results, and previous work identifying that delayed replicative senescence improves anti-CD19 CAR-T cell persistence and function [39], the role of senescence and the DDR merits further exploration in BCMA-targeting and other types of CAR-T cell.

Another observed difference was the expression of the inhibitory receptor LAG-3. In vivo results demonstrated that IL-15 prolonged the persistence of CD8^+^ ARI2h cells that were characterized by low expression of LAG-3 and a CD45RA^-^CD27^+^ central memory phenotype, suggestive of a higher fitness for ARI2h^IL-15^ CARs. Overall, the expression of inhibitory receptors in the ARI2h cells isolated from mice was high, indicating that a combination therapy of ARI2h and checkpoint blockers could act synergistically in the treatment of MM. The loss of CD27 is also associated with CAR-T cell tonic signaling [40], which is another well-known phenomenon that contributes to CAR-T cell dysfunction and the loss of in vivo persistence and tumor-clearing ability [41,42]. Here, we observed that IL-2-grown CAR-T cells exhibited other features of tonic signaling, such as increased cellular size, complexity and DNA content.

In addition to enhancing CAR-T cell tumor clearance and persistence, eliminating or dampening the effect of CRS would be another major improvement to CAR-T cell therapy. In this regard, limiting the production of IFNγ and TNFα by CAR-T cells, which are important CRS-initiating molecules, could be beneficial. Here, we demonstrate that ARI2h^IL-15^ cells, which achieve superior in vivo activity, secrete less IFNγ compared to ARI2h^IL-15/IL-7^ cells. Furthermore, ARI2h^IL-15^ cells produced a lower amount of TNFα, IL-9, SDF-1α and IL-22, all of which are cytokines associated with MM disease progression and/or CAR-T cell-induced toxicity [5,13,35,36,37].

## 4. Materials and Methods

### 4.1. Samples

All donors provided informed written consent in accordance with the Declaration of Helsinki, and all research involving human-derived material was approved by the Ethical Committee of Clinical Research at Hospital Clínic, Barcelona. Buffy coats from healthy donors were obtained from the local blood and tissue bank (Banc de Sang i Teixits, Catalonia). Human T cells were isolated from buffy coats by density-gradient centrifugation using Histopaque-1077 (Sigma-Aldrich, St Louis, MO, USA) followed by negative selection of T cells using a Pan T Cell Isolation Kit (Miltenyi Biotech, Bergisch Gladbach, Germany).

### 4.2. Cell Culture and T Cell Transduction

T cells were stimulated with Dynabeads Human T-Activator CD3/CD28 (Thermo Fisher Scientific, San Diego, CA, USA) and were expanded in Click’s media (47.5% Click’s media (Irvine Scientific, Santa Ana, CA, USA), 47.5% RPMI-1640, 5% human serum, 2 mM L-glutamine, 100 IU/mL penicillin and 100 µg/mL streptomycin) supplemented with 100 IU/mL IL-2 (“IL-2”), 10 ng/mL IL-15 (“IL-15”), 10 ng/mL IL-15 + 10 ng/mL IL-7 (“IL-15/IL-7”) or no cytokine (“None”). At 48 h following stimulation, T cells were transduced with a lentivirus vector encoding ARI2h [17]. Subsequently, T cells were split, and cytokines were refreshed every 1–2 days for a further 7 days of culture, unless indicated otherwise. ARP-1 and U266 cell lines were obtained, cultured and modified to express GFP-firefly luciferase (GFP-ffLuc), as previously described [17]. All cultured cells were incubated at 37 °C with 5% CO_2_. Live cells were counted using Trypan blue exclusion.

### 4.3. Flow Cytometry

Cell surface proteins on CAR-T cells were stained with the following antibodies: CD4-APC/H7, CD8a-PE/Cy7, CCR7-PerCP/Cy5.5, CXCR3-AF488, CD69-FITC, CD3-APC (all from BD Biosciences, San Jose, CA, USA); CD8a-FITC, CD28-FITC, CD27-PE, LAG-3-PE, TIM-3-FITC, TIGIT-PerCP/Cy5.5, CD45RA-APC (all from Biolegend, San Diego, CA, USA); PD-1-APC (eBioscience, Thermo Fisher Scientific, San Diego, CA, USA). To determine CAR expression, cells were labeled with a recombinant Fc-tagged BCMA protein (Enzo Life Sciences, Farmingdale, NY, USA) followed by a BV421-conjugated anti-Fc antibody (Biolegend). Samples prepared from mouse organs were treated with Fc block (BD Biosciences) prior to staining. Cells were washed and resuspended in 1% (*v*/*v*) FCS in PBS or 1% (*v*/*v*) paraformaldehyde prior to acquisition. Live cells were gated based on forward and side scatter.

For intracellular staining of γH2AX, surface-stained cells were fixed in 1% (*v*/*v*) paraformaldehyde, permeabilized in 90% (*v*/*v*) methanol at −20 °C overnight, and then stained with PE-conjugated anti-H2AX (pS139) antibody (BD Biosciences). Immediately prior to analysis, cells were washed and resuspended in 1% (*v*/*v*) FCS in PBS containing 3.3 µg/mL 7-AAD.

All flow cytometry data were acquired on a FACSCanto II (BD Biosciences) and analyzed using FlowJo software, version 7.6.2 (TreeStar, Ashland, OR, USA).

### 4.4. Analysing CAR-T Cell Ribosomal S6 Protein and p38 MAPK Phosphorylation

CAR-T cells were incubated with an equal number of ARP-1 cells, or media, for 6 h at 37 °C, prior to staining with fluorophore-conjugated antibodies. In some cases, CAR-T cells were pretreated with 100 nM rapamycin (Selleckchem, Houston, TX, USA) or 500 nM BIRB-796 (Axon Medchem, Groningen, Netherlands) for 30 min before the assay to act as negative controls for ribosomal S6 protein and p38 MAPK phosphorylation, respectively. Intracellular staining of ribosomal S6 protein and p38 MAPK was carried out as described above for γH2AX, but instead using PE-conjugated anti-S6 (pS235/pS236) and AF647-conjugated anti-p38 MAPK (pT180/pY182) antibodies (both from BD Biosciences).

### 4.5. Degranulation Assay and Granzyme B Expression

CAR-T cells and ARP-1 cells were cultured at a 0.125:1 (degranulation assay) or 1:1 (granzyme B expression) ratio for 6 h at 37 °C. For the degranulation assay, cells were additionally incubated with CD107a-AF647 antibody (BD Biosciences). One hour after the co-culture was initiated, GolgiPlug (1:1000, BD Biosciences) was added to the cells. For intracellular staining of granzyme B, cells were fixed and permeabilized using the FIX&PERM Cell Fixation and Permeabilization Kit (Nordic-MUBio, Susteren, Netherlands) according to the manufacturer’s instructions and stained with an AF647–conjugated anti-granzyme B antibody (BD Biosciences) during the permeabilization step.

### 4.6. SA-β-Gal/Mitochondrial Staining

T-cell SA-β-gal activity was measured using C_12_FDG (Sigma Aldrich) as previously described [43]. For each condition, SA-β-gal activity was calculated as the MFI of C_12_FDG-stained cells, minus the background fluorescence detected in unstained cells. To stain mitochondria and to measure mitochondrial membrane potential, surface-stained cells were incubated with 25 nM TMRM and 10 nM MitoTracker Deep Red FM (both from Thermo Fisher Scientific) for 30 min at 37 °C prior to analysis by flow cytometry.

### 4.7. In Vitro Cytotoxicity Assay

A luminescence-based method, in which the expression of luciferase in cell lines acts as a marker of target cell viability, was employed for all in vitro cytotoxicity assays. For 24-hour assays, 25,000 GFP-ffLuc-expressing ARP-1 (ARP-1-GFP) or U266 (U266-GFP) cells were plated per well of a white 96-well flat-bottomed plate, and T cells were added at the T cell:tumor cell (effector:target) ratios indicated in the figure legends and in triplicates. For long-term challenges, ARP-1-GFP cells were co-cultured with T cells at a 0.125:1 effector:target ratio in a 24-well plate, and 100 µL was transferred to each of triplicate wells of a white 96-well flat-bottomed plate prior to analysis. Just before analyzing luminescence, 100 µg/mL D-luciferin (PerkinElmer, Waltham, MA, USA) was added to the co-cultured cells and the plate was shaken gently for 10 min in the dark. Luminescence measurements were read on a plate reader. The percentage of surviving tumor cells was calculated as 100× (signal from the sample well—background signal)/(signal from the well containing tumor cells alone—background signal).

### 4.8. Long-Term Challenges

T cells were added to ARP-1-GFP cells at a 0.5:1 ratio and after 4 days, 0.5–1 × 10^6^ T cells were analyzed by flow cytometry. Rechallenges were performed by adding T cells from the first challenge to fresh ARP-1-GFP cells at the same 0.5:1 ratio.

### 4.9. Analysis of Cytokine Production

Supernatants collected from a 6-hour co-culture of T cells and an equal number of ARP-1 cells were stored at −80 °C until analysis. The abundance of 34 different cytokines/chemokines was determined using the ProcartaPlex Multiplex Immunoassay Kit (Thermo Fisher Scientific), following the manufacturer’s instructions. Data were analyzed using ProcartaPlex Analyst software (Thermo Fisher Scientific) and a heatmap of the determined protein concentrations was generated using Morpheus software (Broad Institute; https://software.broadinstitute.org/morpheus (accessed on 24 February 2021)). Additionally, levels of IFNγ and IL-2 were measured by ELISA using commercially available kits (Biolegend).

### 4.10. In Vivo Murine Experiments

In vivo mouse experiments were approved by the Ethical Committee of Animal Research (Hospital Clínic, Barcelona, Spain). Mouse experiments were performed as previously described [17]. In brief, irradiated 8-week-old male NOD-SCIDIL2gc^−^/^−^ mice received an intravenous injection of 1 × 10^6^ ARP-1-GFP cells, and 3 weeks later, 8 × 10^6^ UT T cells or 8 × 10^6^ (6 × 10^6^ CAR^+^) CAR-T cells that had been expanded with IL-2, IL-15 or IL-15/IL-7, as described above, were administered to the mice via intravenous injection as well. Thereafter, the bioluminescence signal, corresponding to tumor growth, and the bodyweight of each animal was measured weekly. Beginning 2 weeks after the T-cell infusion, mice were removed from the study when they had a bioluminescence signal that exceeded 20,000 p/sec/cm^2^/sr.

### 4.11. Statistical Analysis

GraphPad Prism version 8.0.1 (GraphPad Prism Software, La Jolla, CA, USA) was used for data analysis. Multiple comparisons were performed using a repeated measures one-way ANOVA, corrected with a Tukey or Dunnett post hoc test. Comparison of mouse survival between different groups was analyzed using the log-rank (Mantel–Cox) test.

## 5. Conclusions

The generation of a rapidly increasing number of new CAR-T cells, especially for hematological malignancies, makes the need to develop improvements to this therapeutic even more timely. Here, we have presented data that could be applied to the development of all types of CAR-T cells, and thus improve treatment protocols for multiple cancers. For BCMA-targeting CAR-T cells in particular, we have demonstrated that a more favorable phenotype and function can be achieved by using IL-15 alone, in place of IL-2 or IL-15/IL-7, during the ex vivo expansion stage. This new finding is a further advancement to a CAR-T cell that is already used in the clinic for R/R MM, and thus has the potential to improve the limited therapeutic options available for these patients.

## Data Availability

Data are available upon reasonable request.

## Supplementary Materials

The following are available online at https://www.mdpi.com/article/10.3390/cancers13143534/s1, Figure S1: Quantification of ARI2h SSC-A (related to Figure 1), Figure S2: IL-15-grown ARI2h BCMA-CARs are highly functional in long-term cytotoxicity assays and short-term generation of functional molecules (related to Figure 2), Figure S3: Further characterisation of ARI2h BCMA-CARs isolated from MM tumour-bearing mice (related to Figure 3), Figure S4: Memory phenotype, ribosomal S6 phosphorylation and CD69 expression of ARI2h BCMA-CARs (related to Figure 4), Figure S5: Expression of TIGIT and CD28 on ARI2h BCMA-CARs (related to Figure 5), Figure S6: Effect of BIRB-796 on p38 MAPK phosphorylation and ARI2h BCMA-CAR DNA damage and mitochondrial phenotype (related to Figure 6), Figure S7: Quantification of IFNγ and IL-2 from multiplex immunoassay experiment (related to Figure 7).
