# Supplementary material for: IL-15 Enhances the Persistence and Function of BCMA-Targeting CAR-T Cells Compared to IL-2 or IL-15/IL-7 by Limiting CAR-T Cell Dysfunction and Differentiation
Source: Cancers (Basel). 2021 Jul 14;13(14):3534. doi: 10.3390/cancers13143534 (PMC8304527; doi:10.3390/cancers13143534)
Supplement: Supplementary file 1 [file cancers-13-03534-s001.zip › cancers-1257078-supplementary.pdf]

Supplementary Materials for

IL-15 Enhances the Persistence and Function of BCMA-targeting CAR-T Cells Compared to IL-2 or IL-15/IL-7 by Limiting CAR-T Cell Dysfunction and Differentiation

Anthony M. Battram, Mireia Bachiller, Victor Lopez, Carlos Fernández de Larrea, Alvaro Urbano-Ispizua, Beatriz Martín-Antonio

Document includes:

Fig. S1. Quantification of ARI2h SSC-A (related to Figure 1).

Fig. S2. IL-15-grown ARI2h BCMA-CARs are highly functional in long-term cytotoxicity assays and short-term generation of functional molecules (related to Figure 2).

Fig. S3. Further characterisation of ARI2h BCMA-CARs isolated from MM tumour-bearing mice (related to Figure 3).

Fig. S4. Memory phenotype, ribosomal S6 phosphorylation and CD69 expression of ARI2h BCMA-CARs (related to Figure 4).

Fig. S5. Expression of TIGIT and CD28 on ARI2h BCMA-CARs (related to Figure 5).

Fig. S6. Effect of BIRB-796 on p38 MAPK phosphorylation and ARI2h BCMA-CAR DNA damage and mitochondrial phenotype (related to Figure 6).

Fig. S7. Quantification of IFNγ and IL-2 from multiplex immunoassay experiment (related to Figure 7).

SUPPLEMENTARY FIGURES

Figure S1

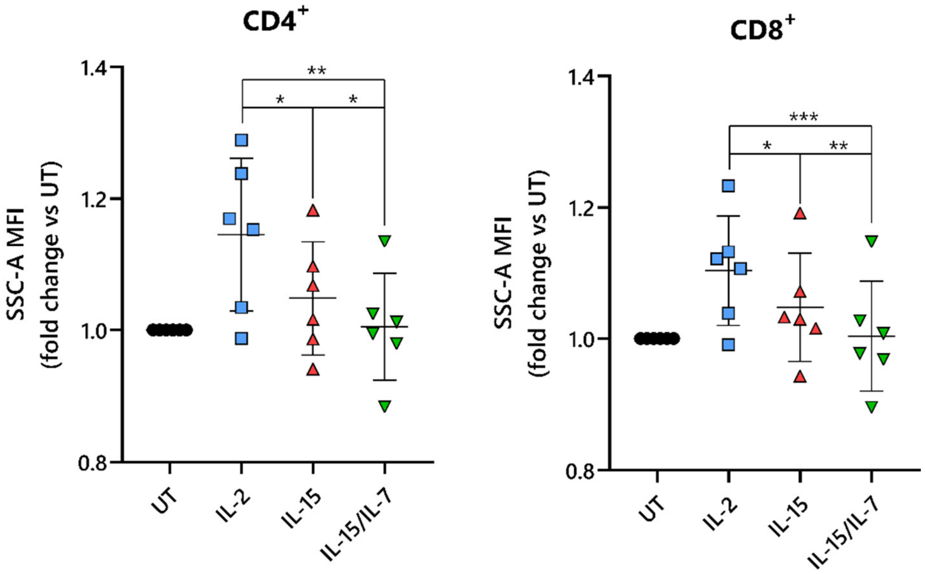

Figure S1. Quantification of ARI2h SSC-A (related to Figure 1). Summary of the SSC-A median fluorescence intensity (MFI) of $CD4^+$ and $CD8^+$ (UT) or BCMA-CAR^+ $CD4^+$ and $CD8^+$ ($ARI2h^{IL-2}$, 'IL-2'; $ARI2h^{IL-15}$, 'IL-15'; $ARI2h^{IL-15/IL-7}$, 'IL-15/IL-7') day 9 *in vitro*-cultured T cells. *, $p<0.05$; **, $p<0.01$; ***, $p<0.001$.

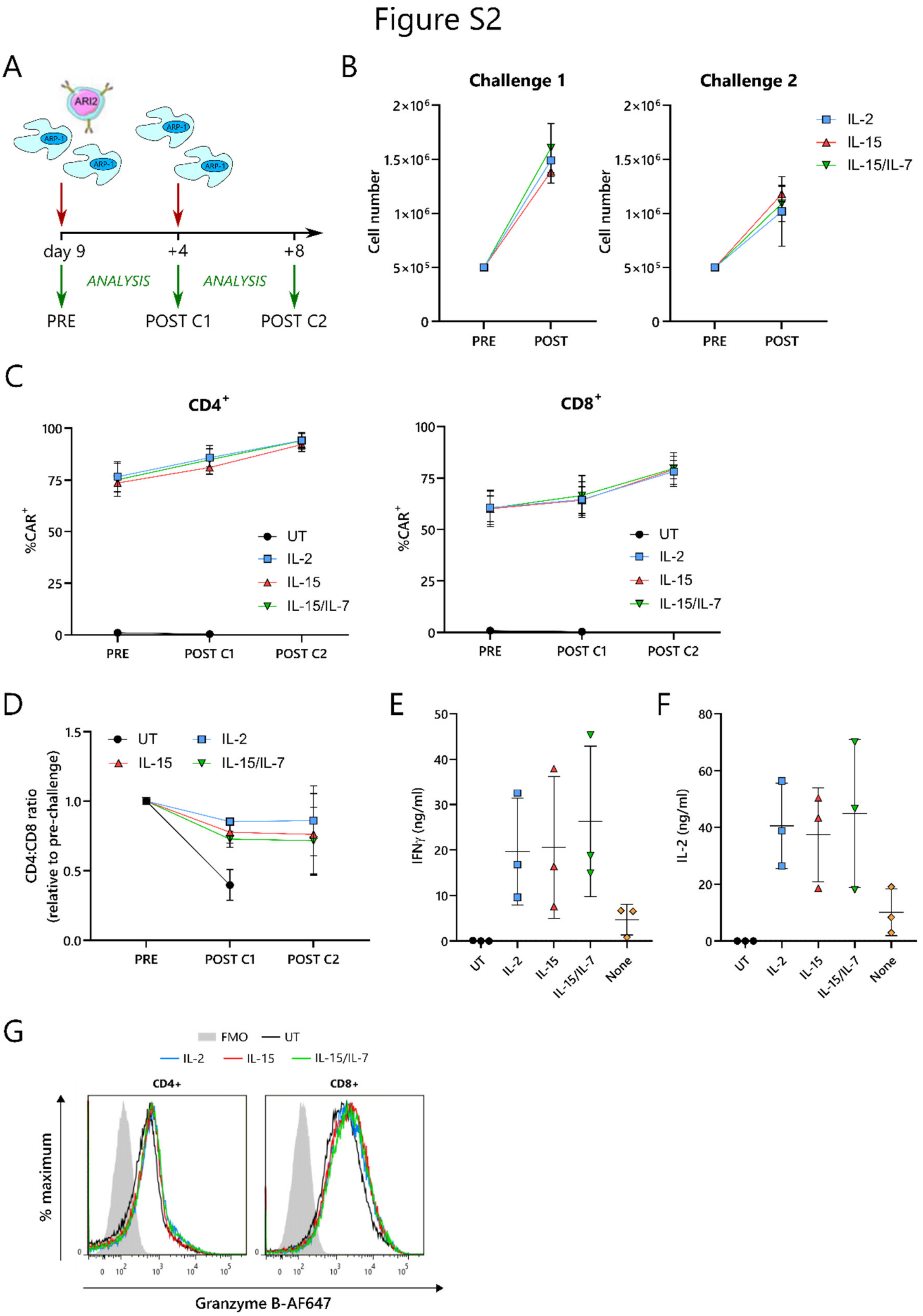

Figure S2. IL-15-grown ARI2h BCMA-CARs are highly functional in long-term cytotoxicity assays and short-term generation of functional molecules (related to Figure 2). A-D) Untransduced (UT) or ARI2h T cells were co-cultured with ARP-1 cells at a 0.5:1 effector:target ratio for 96 hours, before being rechallenged with ARP-1 cells at the same ratio for a further 96 hours. A) Schematic of protocol for multiple challenges of ARI2h cells with ARP-1 cells. B) Cell count of the remaining T cells after the first ('Challenge 1') and the second ('Challenge 2') challenge. C) Percentage of CAR^{+} T cells from UT or ARI2h co-cultures, analysed before (PRE) or after (POST C1) the first challenge, or after the second challenge (POST C2). D)

CD4⁺:CD8⁺ ratio of UT or CAR⁺ T cells, analysed before (PRE) or after (POST C1) the first challenge, or after the second challenge (POST C2) and normalised to PRE samples. E-F) UT and ARI2h T cells were co-cultured with U266 cells for 6 hours and the levels of released IFNγ (E) and IL-2 (F) were measured by ELISA. G) Histograms of granzyme B expression in UT or CAR⁺ CD4⁺ and CD8⁺ day 9 *in vitro*-cultured T cells, representative of 3 experiments. The 'FMO' control shows the staining from UT T cells that were stained with the same antibodies as the 'UT' sample, except the anti-granzyme B antibody.

Figure S3

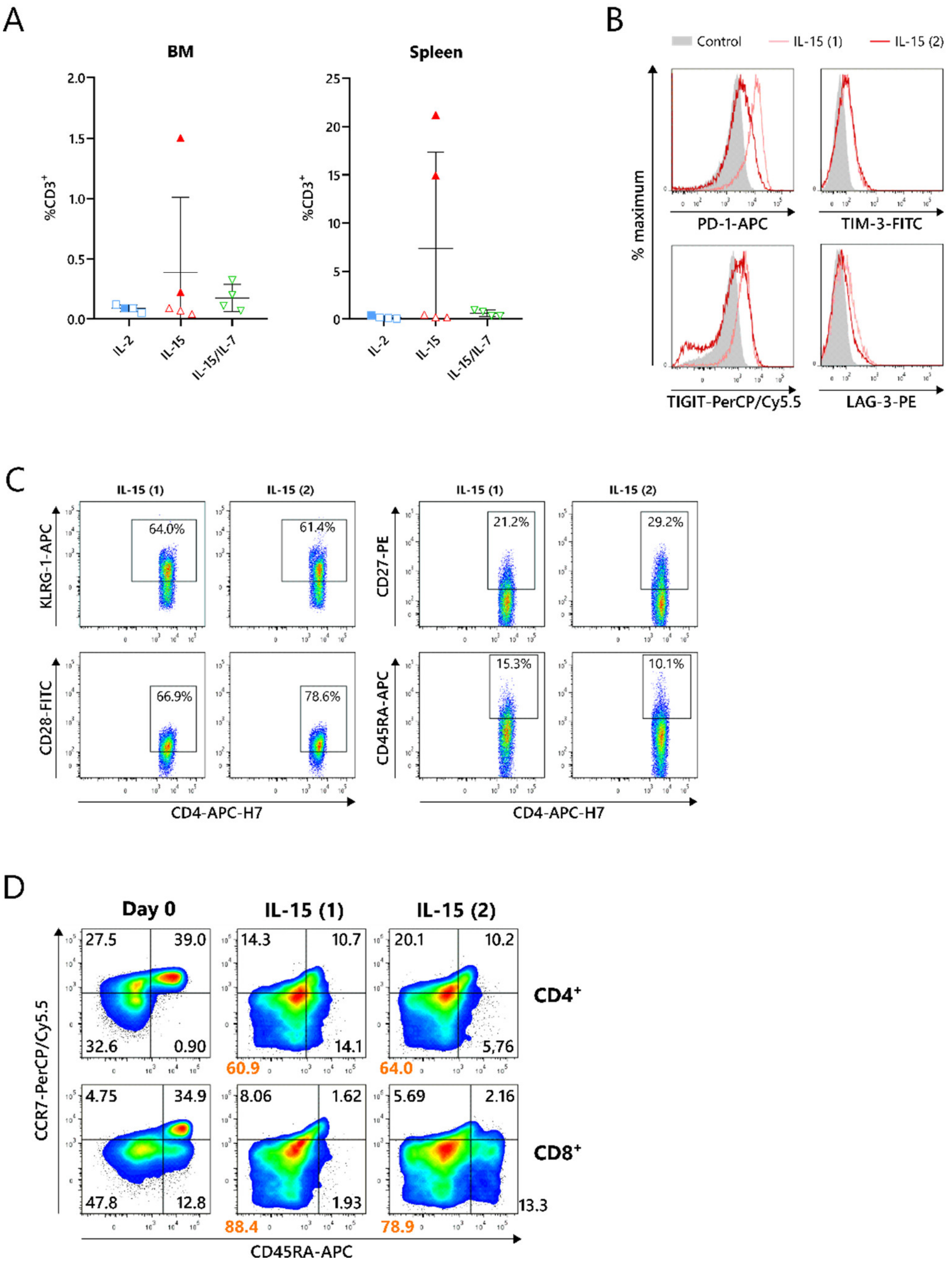

Figure S3. Further characterisation of ARI2h BCMA-CARs isolated from MM tumour-bearing mice (related to Figure 3). A) Frequency of anti-human CD3⁺ ARI2h cells in the bone marrow (BM) and spleen of the non-surviving (open symbols) and surviving (filled symbols) mice from the *in vivo* experiment shown in Figure 3. B-C) Flow cytometry analysis of exhaustion (B) and senescence (C) markers on CD4⁺ ARI2h cells

found in the spleen of the surviving mice from the IL-15 group. ‘Control’ indicates unstimulated human T cells (B). Numbers indicate the percentage of cells found within the gate shown (C). D) Flow cytometry analysis of CCR7 and CD45RA to identify memory/effector cell populations within CD4$^+$ and CD8$^+$ ARI2h cells found in the spleen of the surviving mice from the IL-15 group (IL-15 (1) and IL-15 (2)) and unstimulated CD4$^+$ and CD8$^+$ T cells (Day 0) from the same donor that was used to generate the ARI2h cells that were injected into the mice. Numbers within or next to each quadrant indicate the percentage of cells found within that quadrant.

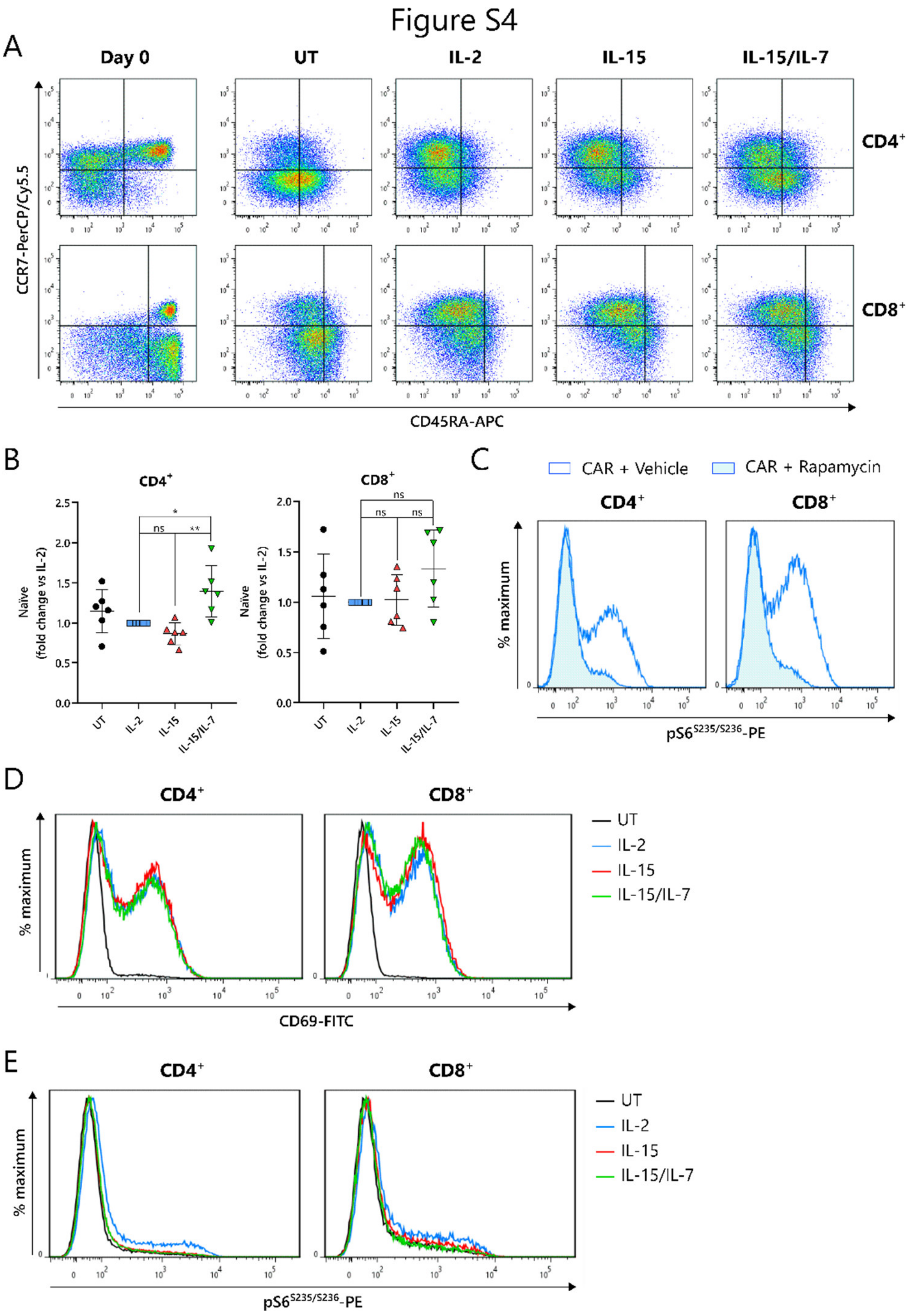

Figure S4. Memory phenotype, ribosomal S6 phosphorylation and CD69 expression of ARI2h BCMA-CARs (related to Figure 4). A) Representative memory phenotype FACS plots from day 0 CD4$^+$ and CD8$^+$ T cells (Day 0) or UT or CAR$^+$ CD4$^+$ and CD8$^+$ day 9 *in vitro*-cultured T cells, representative of 6 experiments. B) Relative frequency of T cells with a naïve phenotype within the CD4$^+$ and CD8$^+$ (UT) or CAR$^+$ CD4$^+$ and CD8$^+$ (ARI2h^{IL-2}, ARI2h^{IL-15}, ARI2h$^{IL-15/IL-7}$) cultures, normalised to ARI2h^{IL-2}. C) Phosphorylation of the ribosomal S6 protein on serine 235/serine 236 in CAR$^+$ CD4$^+$ and CD8$^+$ day 9 ARI2h^{IL-2} cells co-cultured with ARP-1 cells for 6 hours in the presence of 100 nM rapamycin or vehicle control. D) Expression of CD69 on UT or

CAR$^+$ CD4$^+$ and CD8$^+$ T cells after a 6-hour challenge with ARP-1 cells. Shown are representative histograms (n=4). E) Phosphorylation of the ribosomal S6 protein on serine 235/serine 236 in UT or CAR$^+$ CD4$^+$ and CD8$^+$ day 9 *in vitro*-cultured T cells. Shown are representative histograms (n=3). *, p<0.05; **, p<0.01; ns, not significant.

Figure S5

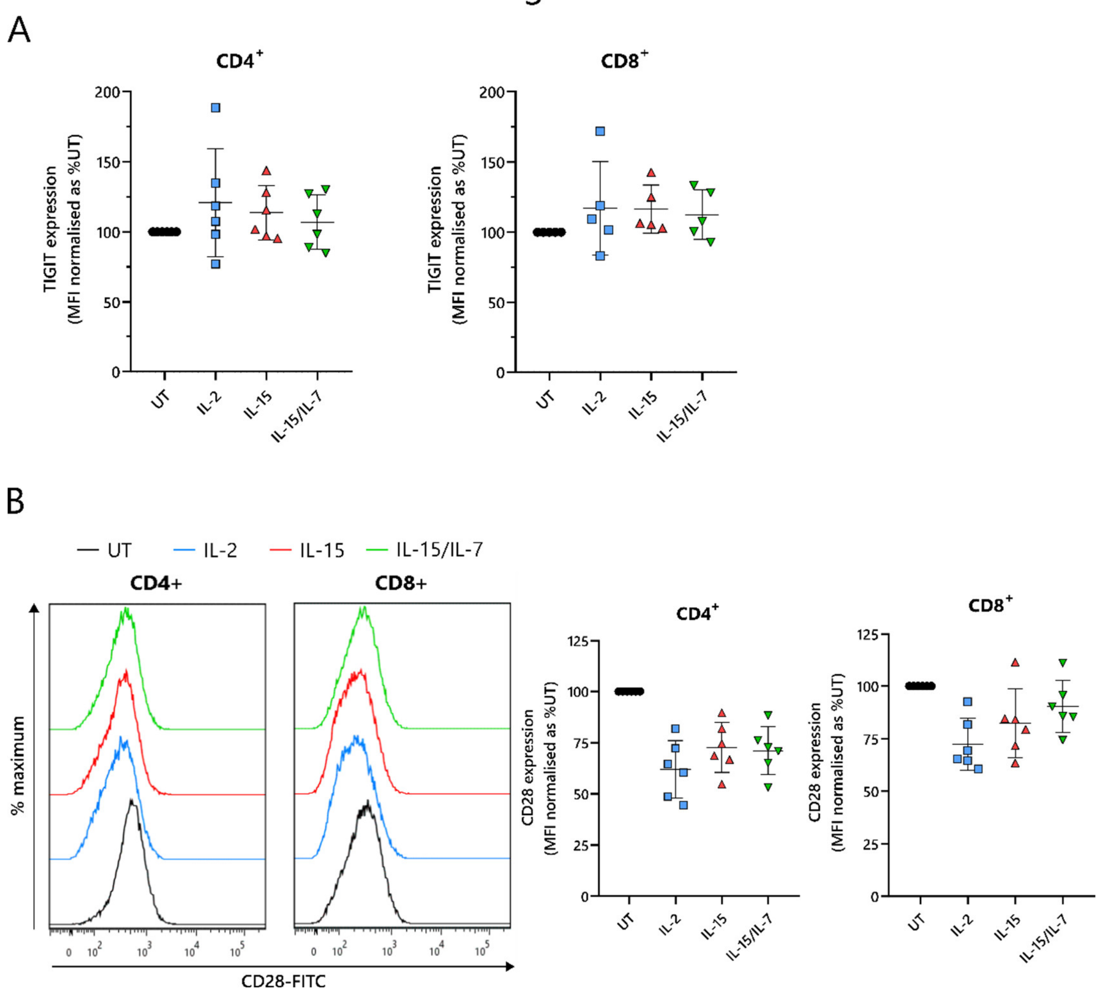

Figure S5. Expression of TIGIT and CD28 on ARI2h BCMA-CARs (related to Figure 5). A) Summary of the surface expression of TIGIT on day 9 *in vitro*-cultured UT or CAR$^+$ CD4$^+$ and CD8$^+$ T cells, based on the MFI of the staining and normalised to UT. B) Left - Representative histograms (left) and summary (right) of the surface expression of CD28 on day 9 *in vitro*-cultured UT or CAR$^+$ CD4$^+$ and CD8$^+$ T cells. Quantification is based on the MFI of the CD28 staining and normalised to UT.

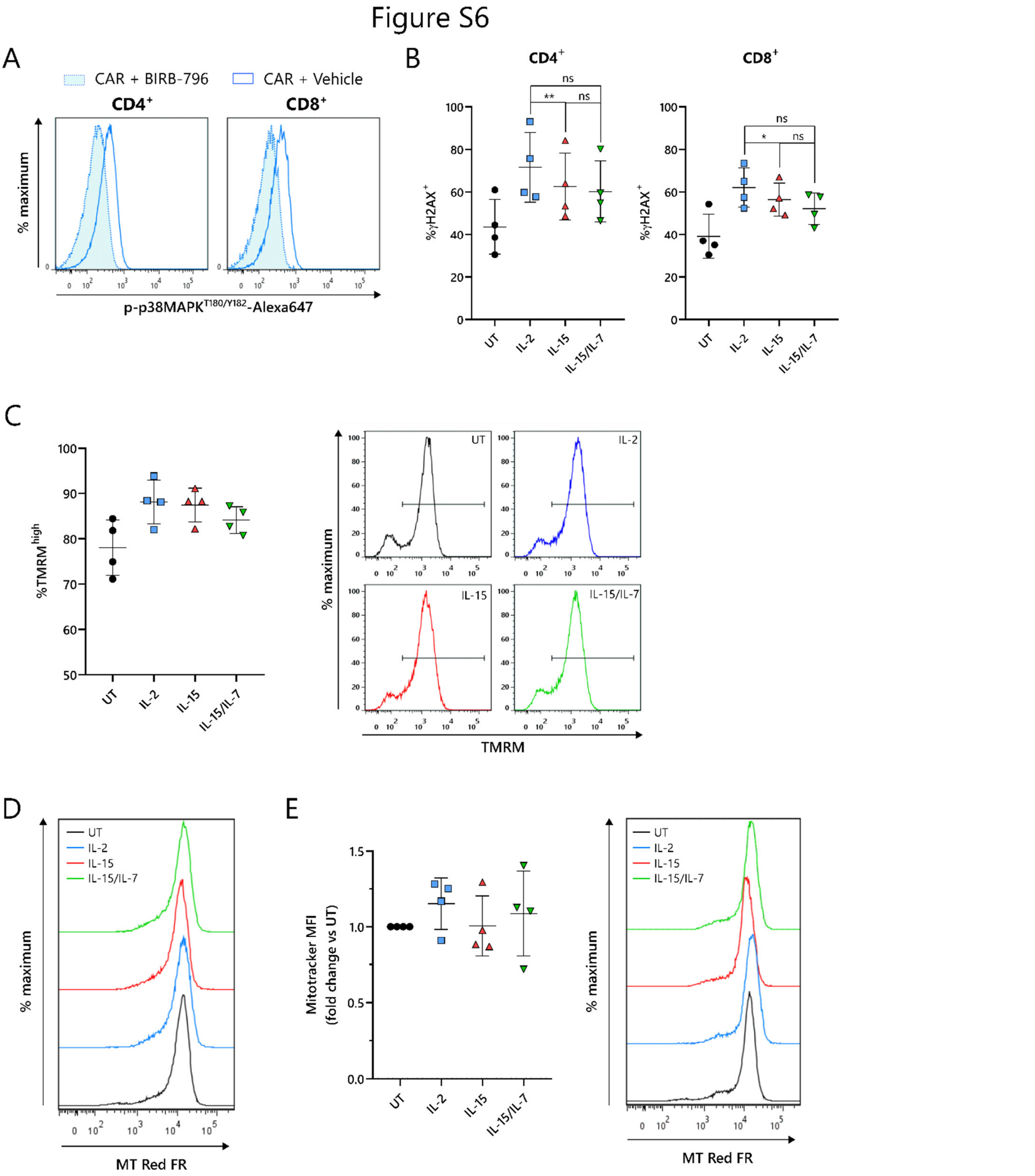

Figure S6. Effect of BIRB-796 on p38 MAPK phosphorylation and ARI2h BCMA-CAR DNA damage and mitochondrial phenotype (related to Figure 6). A) Phosphorylation of p38 MAPK on threonine 180/tyrosine 182 in CAR$^+$ CD4$^+$ and CD8$^+$ day 9 ARI2h^{IL-2} cells treated for 30 minute with 500 nM BIRB-796 or vehicle control. B) Quantification of the percentage of γH2AX$^+$ cells in UT or CAR$^+$ CD4$^+$ and CD8$^+$ day 9 *in vitro*-cultured T cells (related to Figure 6C). C) Left – Relative frequency of hyperpolarised (TMRM^{high}) cells within the populations of CD4$^+$ (UT) or CAR$^+$CD4$^+$ (ARI2h^{IL-2}, ARI2h^{IL-15}, ARI2h$^{IL-15/IL-7}$) day 9 *in vitro*-cultured T cells. Right – Representative histograms (n=4). Gates show hyperpolarised (TMRM^{high}) cells. D-E) Histograms of MitoTracker Deep Red FM (MT Red FR) staining in UT or CAR$^+$ CD8$^+$ (D) and CD4$^+$ (E - right) day 9 *in vitro*-

cultured T cells, representative of 4 experiments. Summary of the CD4$^+$ cell MT Red FR staining based on MFI and normalised to UT (E - left). *, p<0.05; **, p<0.01; ns, not significant.

Figure S7

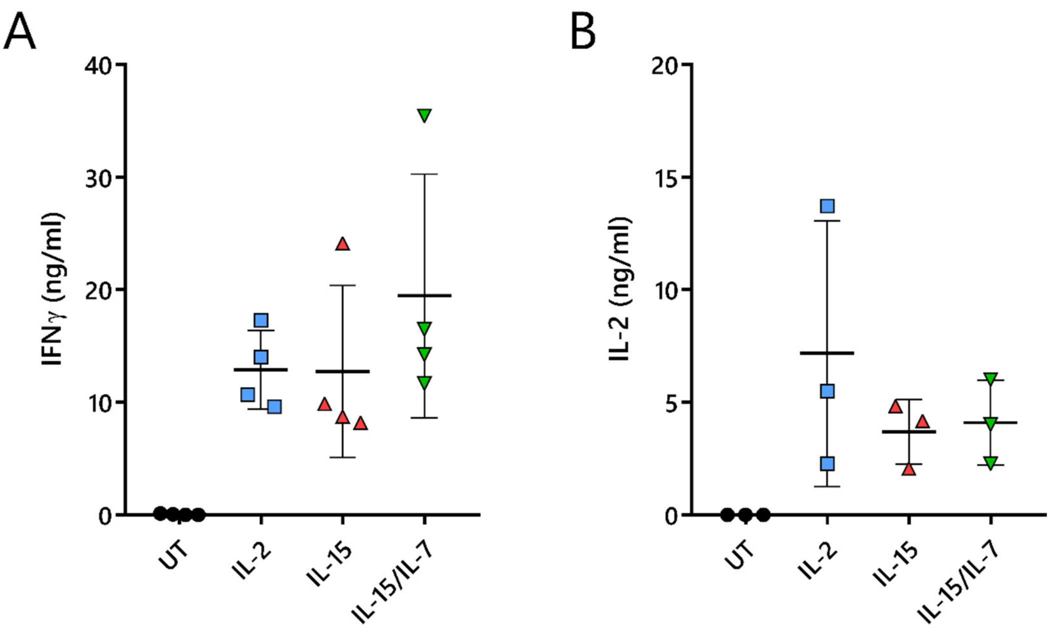

Figure S7. Quantification of IFNγ and IL-2 from multiplex immunoassay experiment (related to Figure 7). Individual quantification of IFNγ (A) and IL-2 (B) levels from the experiment described in Figure 7.
